# Glycaemic, cardiorenal, and lipid parameters associated with SGLT2 inhibitors use in Indonesian patients with type 2 diabetes: 12-month multicenter real-world study

**DOI:** 10.1371/journal.pone.0353564

**Published:** 2026-07-17

**Authors:** Fonny Cokro, Rani Sauriasari, Dicky Levenus Tahapary, Heri Setiawan, Christian Tricaesario, Nurul Hidayati, William Djauhari, Jimmy Tandradynata, Sidartawan Soegondo

**Affiliations:** 1 Clinical and Social Pharmacy Laboratory, Faculty of Pharmacy, Universitas Indonesia, Depok, West Java, Indonesia; 2 Department of Pharmacy, School of Medicine and Health Sciences, Atma Jaya Catholic University of Indonesia, Jakarta, Indonesia; 3 Health Innovation Study and Policy Research Cluster, Faculty of Pharmacy, Universitas Indonesia, Depok, West Java, Indonesia; 4 Division of Endocrinology, Metabolism, and Diabetes, Department of Internal Medicine, Dr. Cipto Mangunkusumo National Referral Hospital, Faculty of Medicine, Universitas Indonesia, Jakarta, Indonesia; 5 Metabolic, Cardiovascular, and Aging Cluster, The Indonesian Medical Education and Research Institute, Faculty of Medicine, Universitas Indonesia, Jakarta, Indonesia; 6 Department of Pharmacology, Faculty of Pharmacy, Universitas Indonesia, Depok, West Java, Indonesia; 7 National Metabolomics Collaborative Research Center, Faculty of Pharmacy, Universitas Indonesia, Depok, West Java, Indonesia; 8 Diabetes Connection and Care, Eka Hospital BSD, South Tangerang, Indonesia; 9 Department of Internal Medicine, Cipto Mangunkusumo National Referral Hospital, Faculty of Medicine, Universitas Indonesia, Jakarta, Indonesia; Mayo Clinic’s Campus in Florida, UNITED STATES OF AMERICA

## Abstract

**Background:**

Sodium-glucose cotransporter-2 inhibitors (SGLT2is) have demonstrated efficacy in glycaemic control and cardiorenal protection across multiple meta-analyses. However, their applicability to the Indonesian population remains uncertain due to demographic, lifestyle, and healthcare disparities. This study aims to evaluate the effectiveness of SGLT2is in managing metabolic control, cardiorenal risk factors, and their safety relative to non-SGLT2is in the T2DM population in Indonesian real clinical settings.

**Methods:**

This study applied a multicenter retrospective cohort design, enrolling adult Indonesian T2DM patients who had received treatment for at least 12 months. Intragroup paired analysis and intergroup comparative studies were performed regarding metabolic control, cardiorenal, and safety parameters.

**Results:**

A total of 638 patients were included, with 319 patients contributing to paired analyses. In paired data analysis, SGLT2is significantly improved HbA1c, fasting plasma glucose (FPG), body weight, body mass index (BMI), systolic blood pressure (SBP), diastolic blood pressure (DBP), lipid profile, and atherosclerotic cardiovascular disease (ASCVD) risk (p < 0.05). Compared to non-SGLT2is, SGLT2is are associated with greater reductions in HbA1c, FPG, body weight, BMI, and SBP. Although no intergroup differences were observed in other parameters, adjusted analyses revealed additional benefits of SGLT2is in lowering low-density lipoprotein (LDL) cholesterol and maintaining estimated Glomerular Filtration Rate (eGFR), though SBP effects became nonsignificant. No major safety signals were observed.

**Conclusion:**

These findings support the use of SGLT2is as effective agents for improving glycaemic control, body weight, blood pressure, lipid profile, and ASCVD risk in Indonesian patients with T2DM. Further studies are warranted to clarify their safety, long-term renal effects, and to explore the comparative effects of individual SGLT2is and dosing strategies.

## Introduction

Indonesia ranks fifth globally in the prevalence of diabetes patients. The population of diabetes patients is expected to rise further [1]. Diabetic cardiorenal complications heighten the likelihood of mortality, raise the risk of being readmitted to the hospital, and diminish patients’ health-related quality of life [2,3]. This concerning tendency requires effective management strategies that can be validated in real-world settings, particularly in specific populations.

Numerous meta-analyses illustrate the effectiveness and safety of Sodium-glucose cotransporter-2 inhibitors (SGLT2is) as medicines for glycaemic control and as cardiorenal protective agents. A meta-analysis shows that SGLT2is significantly diminished Major Adverse Cardiovascular Events (MACE), Hospitalization for Heart Failure (HHF), cardiovascular mortality, and kidney disease progression in individuals with type 2 diabetes mellitus (T2DM) [4]. SGLT2is are linked to decreased blood pressure, weight reduction, and enhanced renal outcomes, which are especially relevant in the context of the high rates of hypertension and chronic kidney disease among diabetes patients [5,6]. Although randomized controlled trials (RCTs) have established the efficacy of SGLT2is in enhancing glycaemic control and mitigating cardiovascular risks [4,7], the direct applicability of findings from large RCTs to the Indonesian population warrants careful consideration, and the relevance of these results to the Indonesian population is questionable due to variations in demographics, lifestyle, and healthcare accessibility [8,9]*.* Differences in clinical characteristics between global and Asian populations may contribute to variations in glycaemic control outcomes. Asian individuals with T2DM often present a distinct clinical phenotype, including younger age at diagnosis, lower body mass index (BMI), and a higher propensity for abdominal adiposity accompanied by reduced muscle mass. This phenotype is associated with an increased risk of insulin resistance and is commonly referred to as the ‘Asian phenotype’ of T2DM [10]. These pathophysiological differences may influence both metabolic responses and safety outcomes associated with glucose-lowering therapies, including SGLT2is. Moreover, despite the overall global generalizability claimed in cardiovascular outcome trials (CVOTs), patients from Southeast Asia remain under-represented in major SGLT2i trials, limiting the robustness of stratified subgroup analyses for this population. Regional and racial subgroup analyses have suggested potential heterogeneity in treatment effects, underscoring the need for population-specific validation [11].

RCTs are conducted under tightly controlled conditions with standardized follow-up and treatment intensification, which do not fully reflect routine clinical practice in Indonesia. Real-world studies can elucidate the efficacy of these drugs in routine clinical settings. Furthermore, to the best of our knowledge, multicentre or comparative studies on this topic in Indonesia are scarce, possibly reflecting limited access to SGLT2is due to financing and availability constraints [8]. This study aims to assess the effectiveness of SGLT2is toward metabolic control and cardiorenal risk factors, along with the safety compared to non-SGLT2is within the Type 2 Diabetes Mellitus (T2DM) population in Indonesia.

## Materials and methods

### Study design and location

A retrospective cohort approach was employed, incorporating data from multiple centers, including a private hospital, a national referral hospital, and a diabetes care facility in Jakarta and its surrounding areas. Retrospective data gathering occurred from July 2024 until May 2025. This study complies with the Declaration of Helsinki, which received approval from the Research Ethics Committee at Cipto Mangunkusumo Hospital (RSCM), reference number KET-744/UN2.F1/ETIK/PPM.00.02/2024 (dated May 24, 2024), and Atma Jaya Catholic University of Indonesia (AJCUI), reference number 01/05/KEP-FKIKUAJ/2024 (dated May 7, 2024). This study’s reporting adhered to the STROBE Checklist for cohort studies. Due to the nature of the retrospective study, the need for informed consent was waived off by the Research Ethics Committee at RSCM and AJCUI. The authors had access to identifiable patient information only during data extraction.

### Study population and exposure

The G*power application calculations indicate that a minimum sample size of 162 participants is necessary for a t-test analysis, utilizing two independent groups, two-tailed, an effect size of d = 0.57, 95% power, and equal group distribution. The effect size d was derived from the findings of the DEFENCE Study, which evaluated HbA1c levels in the dapagliflozin combination group vs a higher dose of single metformin therapy, with mean differences and standard deviations of −0.2 vs. −0.4 and 0.4 vs. 0.3, respectively [12].

This study evaluated two groups: those receiving SGLT2is, either as a combination or as primary diabetes treatment, irrespective of dosage, and those not receiving SGLT2is. The eligibility criteria for this study included individuals with T2DM, aged 18 years or older, regardless of SGLT2is medication status. Diabetes was determined by prior medical diagnosis or the International Classification of Diseases 10 (ICD-10) classification. Dapagliflozin and empagliflozin are the SGLT2 inhibitors currently offered in Indonesia, with their introductions occurring in 2016 and 2017.

We removed any SGLT2is usage of less than 12 months to mitigate confounding. The minimum duration of medication is established at 12 months to evaluate the outcomes due to the results of a meta-analysis indicating that a significant reduction in Urine Albumin-Creatinine Ratio (UACR) can occur within 26–52 weeks of treatment [13], hence providing enough time to demonstrate potential renal protective effects. Incomplete primary outcome data in medical records and discrepancies in data measurement over 24 months were eliminated from this study to mitigate the possibility of bias stemming from patient non-compliance in therapy adherence.

### Variables

This research examines the alterations in HbA1c from the baseline as the primary outcome, while secondary outcomes included alterations in fasting plasma glucose (FPG), systolic blood pressure (SBP), diastolic blood pressure (DBP), body weight, BMI, lipid profile, estimated glomerular filtration rate (eGFR), UACR, Left Ventricular Ejection Fraction (LVEF), and changes in atherosclerotic cardiovascular disease (ASCVD) risk calculated using the revised Pooled Cohort Equations (RPCE) calculator. The RPCE model encompasses age, sex, SBP, antihypertensive therapy history, total cholesterol level, high-density lipoprotein (HDL) cholesterol level, smoking history, and diabetes history; it is preferred for forecasting the incidence of ASCVD over the next decade in Asian populations [14]. This study also evaluated safety, including the incidence of common drug side effects such as UTIs and genital infections, and potential severe side effects, including hypoglycaemia, diabetic ketoacidosis (DKA), fractures, and lower-limb amputations [15]. Adverse events were identified from routine medical record documentation, including physician diagnoses and available laboratory data. A random blood glucose measurement below 70 mg/dL is considered hypoglycaemia; and UTI has been confirmed through urine tests, culture tests, or based on a doctor’s diagnosis. Diabetic ketoacidosis is characterized by ketones in the blood or diagnosed by a physician. Other side effects are determined based on the doctor’s diagnosis.

Several factors may correlate with suboptimal metabolic control and adverse cardiorenal outcomes, potentially confounding this study. These include advancing age, female gender, smoking habits, diabetes duration, multiple current diabetes medications, and coexisting conditions such as hypertension, dyslipidemia, previous cardiovascular events, microvascular issues, and associated side effects [16,17]. The presence of potentially confounding data was assessed retrospectively via the patient’s medical records and presented in the baseline data.

### Statistical analysis

In the data collecting process, missing secondary data concerning lipid profiles was handled using the Sampson-NIH formula, as it is preferred for assessing low-density lipoprotein (LDL), mainly when the LDL value is low (<70 mg/dL), and the triglycerides value is elevated (≥400 mg/dL) [18]. Meanwhile, the missing eGFR data were determined using the CKD-EPI formula to avoid overestimation and serve as a gold standard in estimating eGFR. Additionally, the imputation of missing secondary data was performed with linear interpolation when the baseline data was unavailable, and linear extrapolation when the intermediate or final data was missing. This methodological combination is employed due to its applicability to time series data, mainly when data availability is limited and patterns are discernible. For non-patterned data types, missing data was imputed by computing the average value.

The Kolmogorov-Smirnov test was employed to analyze data normality. The paired t-test was used for paired data when the data distribution was normal, but the Wilcoxon signed-rank test was utilized when the distribution was not normal. Furthermore, the parametric t-test was used for normally distributed data for two independent groups, whereas the Mann-Whitney U test was utilized for non-normally distributed data. The chi-square test was employed for safety components, while Fisher’s exact test was used when the expected cell counts were less than 5. The logistic regression model was used to account for potential confounders, with the median value used as the cut-off point for categorization. The relevant covariates for each outcome were incorporated in this model, including age, gender, and baseline BMI as standard covariates. Given that SGLT2is are advised for an eGFR of 20 mL/min/1.73 m²or above [19], the authors performed a sensitivity analysis by omitting baseline eGFR values below 20 mL/min/1.73 m² and excluding missing data. All statistical analyses were conducted utilizing SPSS Base Version 22 software.

## Results

### Participants flowchart and baseline characteristics

**Fig 1:**
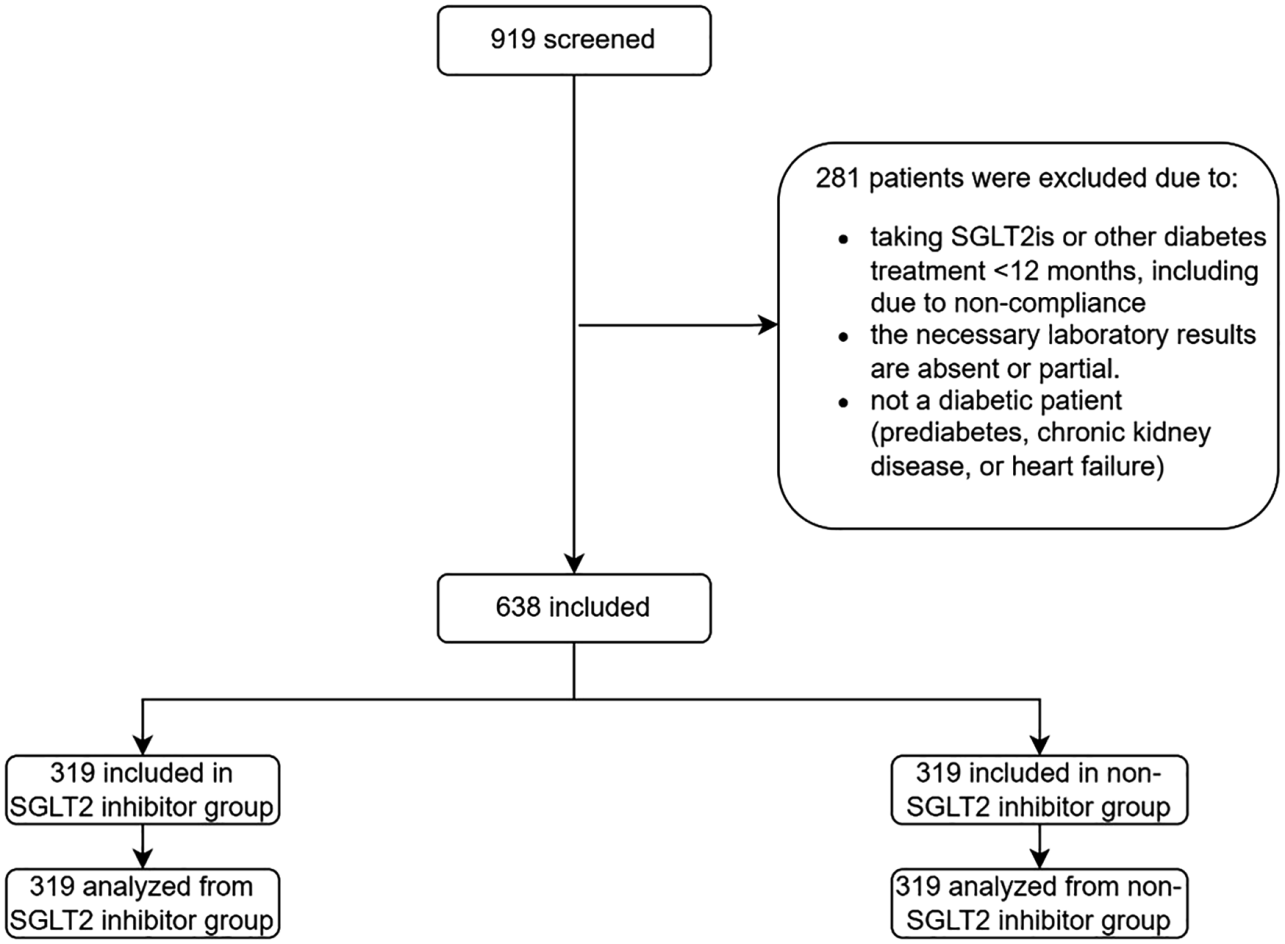
Flow diagram of patients analyzed.

This study screened 919 candidates and included 638 eligible patients. The final study population comprised 319 patients receiving SGLT2is and 319 patients receiving non-SGLT2is, all of whom were included in the comparative analyses between treatment groups (Fig 1). The characteristics of the two groups are deemed statistically comparable, yet disparities exist in some attributes. In the SGLT2is group, the average age was lower, there was a higher proportion of male patients, and patients were subjected to a greater number of diabetes treatments. The mean baseline HbA1c, body weight, BMI, and FPG were higher in the SGLT2is group, suggesting that SGLT2is were mainly utilized as adjunct therapy for patients with uncontrolled diabetes or for those who were overweight or obese. Simultaneously, baseline LDL and HDL cholesterol levels were generally lower in the SGLT2is group. Patients utilizing Glucagon-like peptide-1 (GLP-1) agonists and thiazolidinediones were more prevalent in the SGLT2is group, while the application of acarbose was less common in this group. Patients with a history of diabetic neuropathy, diabetic retinopathy, and hypoglycaemia were predominantly in the non-SGLT2is group. In contrast, those with a history of diabetic nephropathy were primarily in the SGLT2is group. Comprehensive baseline characteristics are included in Table 1.

**Table 1:** Baseline characteristics of patients included in the study.

Characteristic	SGLT2is (N = 319)^a^	Non-SGLT2is (N = 319)^a^	p-value
**Age (years)**	56.64 ± 10.78	58.66 ± 11.81	0.009
**Gender (male)**	189 (59.25%)	153 (48.00%)	0.004
**Smoking history**	22 (6.90%)	27 (8,46%)	0.457
**Diabetes duration (years)**	4 (2–5) (n = 282)	4 (2–4) (n = 292)	0.667
**Number of diabetes medications**	4 (3-4)	2 (2-3)	0.000
**History of Medications:**			
Metformin	259 (81.19%)	257 (80.56%)	0.840
Sulfonylurea	155 (48.59%)	172 (53.92%)	0.178
DPP-4 inhibitor^b^	240 (75.24%)	219 (68.65%)	0.064
GLP-1 agonist^b^	37 (11.60%)	8 (2.51%)	0.000
Metiglinide	0 (0%)	0 (0%)	–
Thiazolidindione	30 (9.40%)	10 (3.13%)	0.001
Acarbose	5 (1.57%)	20 (6.27%)	0.002
**Characteristic**	**SGLT2is (N = 319)** ^**a**^	**Non-SGLT2is (N = 319)** ^**a**^	**p-value**
Insulin	73 (22.88%)	68 (21.32%)	0.633
ACE inhibitor/ ARB^b^	122 (38.13)	124 (38.87)	0.871
Beta-blocker	42 (13.17%)	48 (15.05%)	0.495
Aldosterone antagonist	1 (0.31%)	5 (1.57%)	0.217
Statin	260 (81.50%)	252 (79.00%)	0.426
Aspirin	47 (14.73%)	41 (12.85%)	0.491
**History of Illness:**			
CAD^b^	48 (15.05%)	39 (12.23%)	0.299
Stroke	17 (5.33%)	20 (6.27%)	0.611
PAD^b^	12 (3.76%)	15 (4.70%)	0.550
Hypertension	184 (57.68)	197 (61.76%)	0.294
Dyslipidemia	277 (86.83%)	263 (82.45%)	0.124
Heart failure	7 (2.19%)	15 (4.70%)	0.083
Diabetic nephropathy	57 (17.87%)	32 (10.03%)	0.004
Diabetic neuropathy	28 (8.78%)	59 (18.50%)	0.000
Diabetic retinopathy	12 (3.76%)	29 (9.09%)	0.006
Hypoglycaemia	1 (0.31%)	15 (4.70%)	0.000
HbA1c (% (mmol/mol))^b^	8.82 ± 1.81 (72.90 ± 19.78)	8.21 ± 2.05 (66.23 ± 22.41)	0.000
HbA1c (≤7%)^b^	43 (13.44%)	106 (33.13%)	0.000
Weight (kg)	77.15 ± 15.36 (n = 259)	67.65 ± 13.65 (n = 280)	0.000
BMI^b^	28.61 ± 4.66 (n = 216)	26.50 ± 6.51 (n = 254)	0.000
FPG (mg/dL)^b^	165.86 ± 54.27 (n = 265)	154.16 ± 58.97 (n = 260)	0.000
SBP (mmHg)^b^	133.17 ± 16.87 (n = 256)	130.74 ± 18.07 (n = 282)	0.109
DBP (mmHg)^b^	78.12 ± 8.83 (n = 255)	76.80 ± 9.93 (n = 282)	0.106
LDL (mg/dL)^b^	111.64 ± 42.00 (n = 283)	120.30 ± 47.52 (n = 278)	0.013
HDL (mg/dL)^b^	43.55 ± 11.19 (n = 243)	47.17 ± 14.13 (n = 228)	0.003
TG (mg/dL)^b^	182.26 ± 227.72 (n = 258)	157.73 ± 99.24 (n = 236)	0.050
Total cholesterol (mg/dL)	190.65 ± 51.64 (n = 246)	196.92 ± 55.52 (n = 229)	0.203
eGFR (mL/min/1.73 m^2^)^b^	85.78 ± 26.79 (n = 268)	84.04 ± 30.27 (n = 264)	0.482
ASCVD Risk (%)^b^	13.74 ± 12.26 (n = 199)	13.00 ± 10.54 (n = 203)	0.485

^a^Data in means (SD) or numbers (%) or median (IQR range) for ordinal data type.

^b^DPP-4 inhibitor = Dipeptidyl peptidase 4 inhibitor; GLP-1 agonist = Glucagon-like peptide 1 agonist; ACE inhibitor = Angiotensin-converting enzyme; ARB = Angiotensin receptor blocker; CAD = coronary artery disease; PAD = peripheral artery disease; HbA1c = glycated hemoglobin; BMI = Body mass index; FPG = Fasting plasma glucose; SBP = Systolic blood pressure; DBP = Diastolic blood pressure; LDL = Low-density lipoprotein; HDL = High-density lipoprotein; TG = Triglyceride; eGFR = estimated Glomerulus filtration rate; ASCVD = Atherosclerotic cardiovascular disease.

### Paired data analysis

The administration of SGLT2is considerably enhanced primary and secondary outcomes significantly, except the drop in eGFR, as illustrated in Table 2. These substantial effects were observed after an average exposure length to SGLT2is of 14.18 months. Moreover, sensitivity analysis by excluding baseline eGFR < 20 mL/min/1.73 m^2^ and by excluding missing data yielded significant and robust findings for all outcome components. However, ASCVD risk was no longer significant in the analysis excluding missing data, which included only 18.5% of patients.

**Table 2:** Before-after analysis results of cardiovascular risk parameters.

Components^a^	N	Means ± SD (Baseline)	Means ± SD (After 12 months)	p-value
HbA1c (%)^a^	319	8.82 ± 1.81	7.76 ± 1.35	0.000
Weight (kg)	226	77.57 ± 15.70	76.00 ± 15.23	0.000
BMI^a^	207	29.08 ± 5.10	28.48 ± 4.94	0.000
FPG (mg/dL)^a^	239	164.19 ± 52.22	137.11 ± 38.02	0.000
SBP (mmHg)^a^	220	133.73 ± 17.43	127.39 ± 14.66	0.000
DBP (mmHg)^a^	219	78.29 ± 9.03	76.21 ± 10.16	0.006
LDL (mg/dL)^a^	258	111.46 ± 42.02	96.60 ± 34.73	0.000
HDL (mg/dL)^a^	199	43.37 ± 11.20	44.53 ± 10.95	0.010
TG (mg/dL)^a^	220	185.71 ± 244.96	159.39 ± 109.61	0.016
Total Cholesterol (mg/dL)	202	189.40 ± 51.41	173.44 ± 44.76	0.003
eGFR (mL/min/1.73 m^2^)^a^		84.56 ± 26.09	81.45 ± 26.71	0.000
ASCVD Risk (%)^a^	153	13.05 ± 12.03	11.63 ± 10.99	0.009

^a^HbA1c = glycated hemoglobin; BMI = Body mass index; FPG = Fasting plasma glucose; SBP = Systolic blood pressure; DBP = Diastolic blood pressure; LDL = Low density lipoprotein; HDL = High density lipoprotein; TG = Triglyceride; eGFR = estimated Glomerulus filtration rate; CKD = Chronic kidney disease; ASCVD = Atherosclerotic cardiovascular disease.

### Comparative analysis

SGLT2 inhibitor use was associated with greater reductions in HbA1c compared to non-SGLT2is. Furthermore, patients receiving SGLT2is experienced greater reductions in body weight, BMI, FPG, and SBP compared to non-SGLT2i. Nonetheless, no substantial differences were observed in DBP, LDL, HDL, TG, total cholesterol, eGFR, and ASCVD risk parameters, as illustrated in Table 3. The sensitivity analysis results concerning the eGFR difference, excluding baseline eGFR < 20 mL/minute, were −3.145 mL/min/1.73 m^2^ for the SGLT2is group and −5.637 mL/min/1.73 m^2^ for the non-SGLT2is group; p = 0.061, indicating robustness. The sensitivity analysis, which excluded missing data, demonstrated robust outcomes for all assessed parameters. The availability of comparative outcome data by treatment group is accessible in the supplementary file (S4 Table).

**Table 3:** Assessment of after-treatment and baseline differences between SGLT2is and the comparator.

Components^a^	SGLT2is (mean ± SD)	Non-SGLT2is (mean ± SD)	p-value	N SGLT2is	N Comparator
HbA1c Difference (% (mmol/mol))	−1.051 ± 1.700 (−11.5 ± 18.6)	−0.617 ± 1.977 (−6.7 ± 21.6)	0.000	319	319
Weight Difference (kg)	−1.567 ± 5.100	0.225 ± 4.845	0.000	226	251
BMI Difference	−0.598 ± 1.931	−0.206 ± 3.972	0.001	207	236
FPG Difference (mg/dL)	−27.088 ± 59,574	−6.179 ± 69.926	0.000	239	221
SBP Difference (mmHg)	−6.342 ± 16.648	−1.264 ± 19.842	0.014	220	254
DBP Difference (mmHg)	−2.086 ± 10.500	−1.126 ± 11.322	0.342	219	254
LDL Difference (mg/dL)	−14.865 ± 46.604	−12.018 ± 53.411	0.525	258	243
HDL Difference (mg/dL)	1.157 ± 8.845	0.604 ± 15.218	0.678	199	168
TG Difference (mg/dL)	−26.321 ± 220.850	−22.257 ± 93.163	0.435	220	172
Total Cholesterol Difference (mg/dL)	−15.959 ± 59.743	−12.814 ± 63.485	0.624	202	168
eGFR Difference (mL/min/1.73 m^2^)	−3.111 ± 12.776	−5.624 ± 14.710	0.057	225	211
ASCVD Risk Difference (%)	−1.418 ± 7.027	0.038 ± 6.146	0.059	153	145

^a^HbA1c = glycated hemoglobin; BMI = Body mass index; FPG = Fasting plasma glucose; SBP = Systolic blood pressure; DBP = Diastolic blood pressure; LDL = Low density lipoprotein; HDL = High density lipoprotein; TG = Triglyceride; eGFR = estimated Glomerulus filtration rate; ASCVD = Atherosclerotic cardiovascular disease

### Safety profile

Concerning safety issues, there were no significant differences between the two groups in the incidence of genital infections, urinary tract infections (UTIs), hypoglycaemia, fractures, lower-limb amputations, or any adverse events (p > 0.05), as seen in Table 4. No cases of genital infections or diabetic ketoacidosis were identified based on available medical records.

**Table 4:** Assessment of safety between SGLT2is and the comparator.

Components	n SGLT2is	n Comparator	p-value	N SGLT2is	N Comparator
Genital infections	0	0	–	319	319
Urinary tract infections	9	12	0.51	319	319
Hypoglycaemia	1	2	1.00	319	319
Diabetic ketoacidosis	0	0	–	319	319
Fractures	2	1	1.00	319	319
Lower-limb amputation	0	2	0.50	319	319
Any adverse events	12	16	0.44	319	319

### Logistic regression analysis

**Fig 2:**
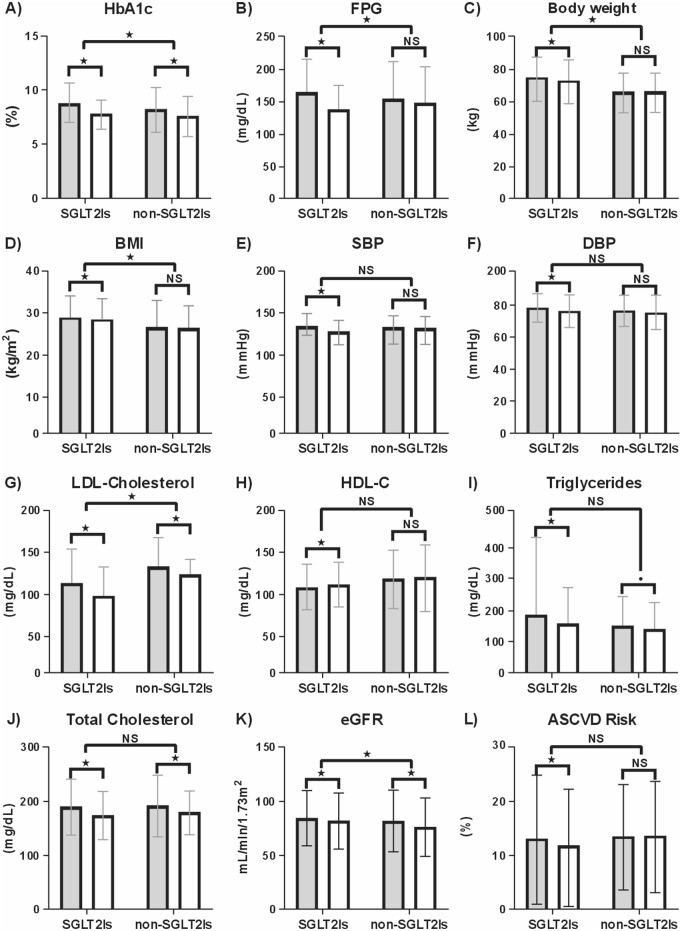
Paired data and comparative analysis results of cardiovascular risk parameters after adjustments. (*) = significant; NS = non-significant; grey bar represents the baseline state, white bar indicates the assessment following a minimum of 12 months of therapy; HbA1c = glycated hemoglobin; BMI = Body mass index; FPG = Fasting plasma glucose; SBP = Systolic blood pressure; DBP = Diastolic blood pressure; LDL = Low density lipoprotein; HDL = High density lipoprotein; eGFR = estimated Glomerulus filtration rate; ASCVD = Atherosclerotic cardiovascular disease.

This analysis did not alter the significance of the results in each component, except for the difference in SBP between groups, which became insignificant after adjusting for covariates such as age, gender, history of coronary artery disease (CAD), history of stroke, history of diabetic nephropathy, baseline BMI, and baseline SBP. Simultaneously, the disparity in LDL and eGFR between the groups was advantageous for the SGLT2is group; following adjustments for age, gender, diabetes duration, dyslipidemia history, baseline BMI, and baseline LDL for the LDL difference (OR = 2.070; 95%CI = 1.255–3.414; p = 0.004); and for age, gender, the quantity of antidiabetic agents, baseline BMI, and baseline eGFR for the eGFR difference (OR = 1.803; 95%CI = 1.048–3.100; p = 0.033). All other parameters yielded robust conclusions as the comparative analysis after controlling for confounders, including: HbA1c difference (OR = 1.855; 95%CI = 1.083–3.179; p = 0.024), FPG difference (OR = 2.000; 95%CI = 1.070–3.739; p = 0.030), body weight difference (OR = 1.756; 95%CI = 1.191–2.589; p = 0.004), BMI difference (OR = 1.606; 95%CI = 1.068–2.415; p = 0.023), DBP difference (OR = 1.345; 95%CI = 0.862–2.098; p = 0.192), HDL difference (OR = 1.229; 95% = 0.746–2.025; p = 0.418), triglycerides difference (OR = 0.722; 95%CI = 0.433–1.206; p = 0.214), total cholesterol difference (OR = 1.131; 95%CI = 0.643–1.990; p = 0.668), and ASCVD risk difference (OR = 1.425; 95%CI = 0.837–2.427; p = 0.192). For the eGFR difference, a cut-off point of 0 was employed instead of the median value, owing to the median’s negative value. The full logistic regression output is available in the supplementary file (S5 Table). The summary of the findings are presented in Fig 2.

## Discussion

The paired analysis showed favorable changes in glycaemic control and cardiovascular risk parameters among patients receiving SGLT2is in the Indonesian population. In this study, a drop of HbA1c of 1.05% (11.48 mmol/mol) is deemed clinically significant, whereas a 1% (10.93 mmol/mol) decrease corresponds to a 21% reduction in diabetes-related mortality [20]. Likewise, a minimum weight reduction of 0.45–4.08 kg in overweight T2DM patients reduces mortality rates [21]. The findings of this investigation align with a notable RCT conducted by Kaku et al. that investigated the effects of tofogliflozin as monotherapy in Japanese patients with T2DM. The results indicated significant reductions in HbA1c levels, body weight, and blood pressure during therapy, implying the drug’s effectiveness in improving glycaemic control and reducing cardiovascular risk factors [22]. Meanwhile, the advantageous effects of SGLT2is on cardioprotection enhancement involve various mechanisms, such as modulation of sympathetic activity, enhancement of fatty acid utilization as an energy source [23], amelioration of insulin resistance, enhancement of endothelial function, and facilitation of natriuresis, resulting in diminished sodium concentrations in muscle tissue [24].

Compared to non-SGLT2is, the primary outcome measurement for glycaemic control, indicated by HbA1c, aligned with a meta-analysis conducted in broader population comparing the effects of SGLT2is and sulfonylureas, which revealed that the SGLT2is exhibited greater HbA1c improvement than sulfonylurea treatment combined with metformin [25]. Numerous trials have demonstrated comparable outcomes favoring SGLT2is over DPP-4 inhibitors in reducing HbA1c levels [26]. This recent real-world investigation revealed that, after controlling for confounding variables, the use of SGLT2is was associated with a 1.855-fold increased likelihood of lowering HbA1c by 0.5% compared to non-SGLT2is. A meta-analysis encompassing 12 CVOTs indicated that a 0.5% reduction in HbA1c lowered the MACE Hazard Ratio by 20% [27]. The outcomes of this study reinforce the evidence for the efficacy of SGLT2is compared to alternative antidiabetic medications in attaining glycaemic control, especially among diabetic patients in developing countries.

The results of this real-world study corroborated earlier findings about weight loss [25]. Regarding blood pressure, after controlling for confounding variables, the advantages of SGLT2is were comparable to those of non-SGLT2is. However, compared to baseline values, SGLT2is markedly decreased blood pressure, both statistically and clinically. A meta-analysis of 48 randomized controlled trials indicated that a 5 mmHg decrease in SBP correlates with a 10% reduction in cardiovascular disease events [28], highlighting the significance of SGLT2is in enhancing metabolic control and mitigating cardiorenal risk.

The administration of SGLT2is indicated to enhance LDL, although it did not exhibit statistically significant differences between groups on HDL, triglyceride, and total cholesterol. A recent meta-analysis encompassing 60 RCTs with 147,130 participants demonstrated that SGLT2is elevated total cholesterol, increased LDL cholesterol, augmented HDL cholesterol, and decreased triglycerides. This study is limited by significant heterogeneity, potentially arising from differences in fundamental variables, including gender, age, BMI, ethnicity, type of SGLT2is, therapeutic indication, duration, and dosage. Particularly concerning LDL, the existence of distinct kinds based on size and density contributes to heterogeneity [29].

Sensitivity analyses, both including and excluding prior statin use in the logistic regression models, showed that LDL reduction remained significant in the SGLT2 inhibitor group, suggesting an association independent of statin use. However, the observational design limits causal interpretation. Additional prospective and biomarker studies are required to evaluate the specific types of SGLT2is and their dosages on different elements of the lipid profile.

This investigation revealed a beneficial cardioprotective benefit SGLT2is relative to baseline values; however, the reduction in the risk of ASCVD was not statistically significant when compared to non-SGLT2is. A meta-analysis indicated that SGLT2is do not diminish the occurrence of MACE in Asian subpopulations [30], but another meta-analysis of CVOTs shown significant cardiorenal enhancements in the general population [4]. Moreover, prior CVOTs exhibited enhanced cardiovascular results over a duration of 3.1 to 4.2 years, especially in diabetic individuals at elevated ASCVD risk [31]. Consequently, it is essential to perform real-world studies involving prolonged utilization of SGLT2is.

Regarding renal parameters, eGFR was observed to decline in both groups. However, logistic regression analysis indicated that SGLT2is use was associated with a lower likelihood of eGFR decline. Prior clinical trials showed a modest decline in eGFR within 3–6 weeks, induced by osmotic diuretics following the initiation of SGLT2is, succeeded by a phase of stable eGFR lasting from 52 to 104 weeks [32]. A global meta-analysis demonstrated that the renal protective effect of SGLT2is, via improvement of renal disease progression, is observable at a median follow-up of 1.3 to 2.6 years [31]. Nevertheless, the present study only included a 12-month observation period and did not evaluate longitudinal eGFR slopes. Therefore, it is not possible to determine whether the observed decline in eGFR reflects the expected acute hemodynamic response to SGLT2is therapy or progressive renal function deterioration. Consequently, real-world research lasting two years or more is required to evaluate the long-term impact of SGLT2is on eGFR.

SGLT2 inhibitors appear to be safe, with few reported adverse events; however, potential underreporting in medical records and the low event frequency limit the statistical power to detect meaningful between-group differences. Numerous prior studies indicate that the use of SGLT2is elevates the occurrence of genital infections by 1.86 to 4.17 times relative to other antidiabetic treatments [33]. Nonetheless, differences in post-defecation hygiene practices were found between Asians and Westerners [34]. Consequently, these behavioral differences may obscure the detrimental effects of consuming SGLT2is in Asia.

This multicenter study encompasses primary to tertiary care settings and includes both government and private hospitals, allowing for the capture of diverse patient characteristics. However, this study possesses multiple drawbacks. The primary limitation of this study is its retrospective design, which may introduce biases, including immortal time bias and missing data. Nevertheless, the final power of 97.94% from OpenEpi primary outcome calculation, indicates that the sample size remains enough to analyze the outcomes. The use of linear interpolation and extrapolation for missing data assumes linear trends over time and may not fully capture non-linear variations, particularly for parameters such as lipid profiles. This may introduce bias compared to more advanced imputation methods. Nevertheless, sensitivity analyses of comparative data indicate that the main findings remained robust despite the imputation approach used. The second limitation is the significant imbalance in several baseline characteristics between groups. The higher baseline HbA1c observed in the SGLT2is group may partially explain the greater absolute reductions in glycaemic parameters, as higher initial values are generally associated with larger subsequent declines. In contrast, the lower baseline HbA1c in the comparator group may have limited the extent of further improvement, suggesting a potential ceiling effect. Furthermore, the treatment assignment model demonstrated good discrimination (c-statistic [AUC] = 0.836) and explained a substantial proportion of treatment allocation variability (Nagelkerke R² = 0.422), indicating that measured baseline characteristics strongly influenced the likelihood of receiving SGLT2is. This pattern may reflect preferential prescribing of SGLT2is to patients with more severe hyperglycaemia and higher cardiometabolic risk, as recommended by various guidelines [35,36]. Although multivariable adjustment was performed, residual confounding related to both measured and unmeasured baseline differences cannot be fully excluded. The third limitation is the inability to accurately assess medication adherence because of incomplete data, potentially leading to underestimated findings. The fourth barrier pertains to the limited recommendations from clinicians for doing UACR and LVEF exams, rendering the acquisition of these data challenging and unanalyzable. Similarly, adverse effects may remain unrecorded. The fifth limitation is the unquantifiable dosage variation during therapy, which occurs multiple times annually in numerous patients. Detailed information on SGLT2 inhibitor dosing and dose adjustments over time was not consistently available. These factors may produce variability that could not be fully accounted for in this study. Additionally, the use of median cut-offs to dichotomize continuous variables may have reduced statistical power and obscured clinically meaningful differences. Future analyses using continuous modeling are warranted.

## Conclusion

In conclusion, SGLT2 inhibitors were associated with improvements in glycaemic control, body weight, blood pressure, lipid profile, and ASCVD risk in Indonesian patients with T2DM. No major safety signals were observed; however, the findings regarding renal parameters and safety outcomes should be interpreted with caution. Further studies are warranted to evaluate safety, long-term renal outcomes, and comparative effects across different SGLT2 inhibitors and dosing strategies.

## Supporting information

S1 Table Dataset. (DOCX)

S2 Table Sensitivity analyses results. (DOCX)

S3 Table STROBE checklist. (DOC)

S4 Table Availability of comparative outcome data by treatment group. (DOCX)

S5 Table Full logistic regression output. (DOCX)
