# Supplementary material for: Glycaemic, cardiorenal, and lipid parameters associated with SGLT2 inhibitors use in Indonesian patients with type 2 diabetes: 12-month multicenter real-world study
Source: PLoS One. 2026 Jul 17;21(7):e0353564. doi: 10.1371/journal.pone.0353564 (PMC13378966; doi:10.1371/journal.pone.0353564)
Supplement: S1 Table — (DOCX) [file pone.0353564.s001.docx]

**S1. Dataset**

Title: “SGLT2 inhibitors improve glycaemic, cardiorenal, and lipid parameters: 12-month evidence from Indonesia”.

The dataset can be accessed through figshare ([doi: 10.6084/m9.figshare.32038683](https://doi.org/10.6084/m9.figshare.32038683)).
