# Supplementary material for: Glycaemic, cardiorenal, and lipid parameters associated with SGLT2 inhibitors use in Indonesian patients with type 2 diabetes: 12-month multicenter real-world study
Source: PLoS One. 2026 Jul 17;21(7):e0353564. doi: 10.1371/journal.pone.0353564 (PMC13378966; doi:10.1371/journal.pone.0353564)
Supplement: S2 Table — (DOCX) [file pone.0353564.s002.docx]

**S2. Sensitivity analyses results**

- Paired data

| **Components^a^** | **Baseline means** | **Baseline SD** | **Means after 12 months** | **SD after 12 months** | **N SGLT2is** | **p-value** |
| --- | --- | --- | --- | --- | --- | --- |
| **Results of the sensitivity analyses after excluding baseline eGFR <20 mL/minute** | | | | | | |
| e-GFR | 85,162 | 25,408 | 82,017 | 26,135 | 223 | 0,000 |
| **Results of the sensitivity analyses after excluding imputed missing data** | | | | | |  |
| Bosy weight | 77,568 | 15,655 | 75,975 | 15,163 | 221 | 0,000 |
| BMI | 29,062 | 5,125 | 28,454 | 4,963 | 202 | 0,000 |
| FPG | 165,550 | 53,245 | 137,442 | 39,066 | 222 | 0,000 |
| SBP | 133,290 | 17,364 | 127,260 | 14,831 | 213 | 0,000 |
| DBP | 78,130 | 8,968 | 76,030 | 10,245 | 212 | 0,007 |
| LDL | 110,330 | 41,227 | 96,190 | 34,622 | 246 | 0,000 |
| HDL | 43,550 | 11,365 | 44,640 | 11,081 | 188 | 0,017 |
| TG | 187,670 | 252,692 | 160,740 | 111,831 | 206 | 0,025 |
| Total cholesterol | 187,700 | 53,541 | 168,210 | 48,002 | 92 | 0,035 |
| e-GFR | 84,825 | 26,386 | 81,076 | 26,773 | 215 | 0,000 |
| ASCVD risk | 12,853 | 12,513 | 11,827 | 12,982 | 59 | 0,069 |

**^a^**BMI = Body mass index; FPG = Fasting plasma glucose; SBP = Systolic blood pressure; DBP = Diastolic blood pressure; LDL = Low density lipoprotein; HDL = High density lipoprotein; TG = Triglyceride; eGFR = estimated Glomerulus filtration rate; ASCVD = Atherosclerotic cardiovascular disease

- **Comparative analysis**

| **Components^a^** | **SGLT2 is** | **Comparator** | **N SGLT2is** | **N Comparator** | **p-value** |
| --- | --- | --- | --- | --- | --- |
| **Results of the sensitivity analyses after excluding baseline eGFR <20 mL/minute** | | | | | |
| e-GFR difference | -3,145 ± 12,828 | -5,637 ± 14,744 | 223 | 210 | 0,061 |
| **Results of the sensitivity analyses after excluding imputed missing data** | | | | | |
| Body weight difference | -1,592 ± 5,153 | 0,225 ± 4,845 | 221 | 251 | 0,000 |
| BMI difference | -0,608 ± 1,952 | -0,206 ± 3,972 | 202 | 236 | 0,001 |
| FPG difference | -28,103 ± 59,875 | -5,804 ± 70,056 | 222 | 219 | 0,000 |
| SBP difference | -6,030 ± 16,604 | -1,260 ± 19,842 | 213 | 254 | 0,025 |
| DBP difference | -2,100 ± 10,582 | -1,130 ± 11,322 | 212 | 254 | 0,339 |
| LDL difference | -14,138 ± 46,233 | -12,018 ± 53,411 | 246 | 243 | 0,639 |
| HDL difference | 1,085 ± 8,953 | 0,886 ± 15,647 | 188 | 164 | 0,882 |
| TG difference | -26,34 ± 226,942 | -10,650 ± 93,432 | 207 | 167 | 0,403 |
| Total cholesterol difference | -17,080 ± 69,231 | -0,650 ± 60,683 | 93 | 102 | 0,079 |
| e-GFR difference | -3,769 ± 12,172 | -5,624 ± 14,710 | 212 | 211 | 0,158 |
| ASCVD difference | -1,216 ± 6,236 | 0,148 ± 6,827 | 62 | 84 | 0,218 |

**^a^**BMI = Body mass index; FPG = Fasting plasma glucose; SBP = Systolic blood pressure; DBP = Diastolic blood pressure; LDL = Low density lipoprotein; HDL = High density lipoprotein; TG = Triglyceride; eGFR = estimated Glomerulus filtration rate; ASCVD = Atherosclerotic cardiovascular disease
