# Supplementary material for: Glycaemic, cardiorenal, and lipid parameters associated with SGLT2 inhibitors use in Indonesian patients with type 2 diabetes: 12-month multicenter real-world study
Source: PLoS One. 2026 Jul 17;21(7):e0353564. doi: 10.1371/journal.pone.0353564 (PMC13378966; doi:10.1371/journal.pone.0353564)
Supplement: S4 Table — (DOCX) [file pone.0353564.s004.docx]

**S4. Availability of comparative outcome data by treatment group**

| **Outcome** | **Baseline Available (n(%))** | | **Follow-up Available (n(%))** | | **Missing (n(%))** | | **Imputated for Missing Data (n(%))^a^** | |
| --- | --- | --- | --- | --- | --- | --- | --- | --- |
|  | **N SGLT2is** | **N Comparator** | **N SGLT2is** | **N Comparator** | **SGLT2is** | **Comparator** | **SGLT2is** | **Comparator** |
| HbA1c | 316 (99.06) | 319 (100) | 311 (97.49) | 319 (100) | 10 (3.13) | 0 (0) | 10 (3.13) | 0 (0) |
| FPG | 257 (80.56) | 259 (81.19) | 262 (82.13) | 246 (77.12) | 97 (30.41) | 100 (31.35) | 17 (5.33) | 2 (0.63) |
| Body weight | 259 (81.19) | 280 (87.77) | 258 (80.88) | 276 (86.52) | 98 (30.72) | 68 (21.32) | 5 (1.57) | 0 (0) |
| BMI | 236 (73.98) | 269 (84.33) | 238 (74.61) | 260 (81.50) | 117 (36.68) | 83 (26.02) | 5 (1.57) | 0 (0) |
| SBP | 256 (80.25) | 282 (88.40) | 248 (77.74) | 281 (88.09) | 106 (33.23) | 65 (20.38) | 7 (2.19) | 0 (0) |
| DBP | 255 (79.94) | 282 (88.40) | 248 (77.74) | 281 (88.09) | 107 (33.54) | 65 (20.38) | 7 (2.19) | 0 (0) |
| LDL-C | 279 (87.46) | 278 (87.47) | 270 (84.64) | 265 (83.07) | 73 (22.88) | 75 (23.51) | 12 (3.76) | 0 (0) |
| HDL-C | 240 (75.24) | 227 (71.16) | 219 (68.65) | 204 (63.01) | 130 (40.75) | 152 (47.65) | 10 (3.13) | 1 (0.31) |
| Triglycerides | 251 (78.68) | 236 (73.98) | 241 (75.55) | 212 (66.46) | 111 (34.80) | 134 (42.01) | 12 (3.76) | 0 (0) |
| Total cholesterol | 242 (75.86) | 229 (71.79) | 224 (70.22) | 206 (64.58) | 127 (39.81) | 152 (47.65) | 10 (3.13) | 1 (0.31) |
| e-GFR | 261 (81.82) | 263 (82.46) | 240 (75.24) | 233 (73.04) | 108 (33.86) | 108 (33.86) | 14 (4.39) | 0 (0) |
| ASCVD risk | 193 (60.50) | 203 (63.64) | 178 (55.80) | 182 (57.05) | 178 (55.80) | 174 (54.55) | 13 (4.08) | 1 (0.31) |

**^a^**Missing data imputation based on supporting data availability

**^b^**HbA1c = glycated hemoglobin; BMI = Body mass index; FPG = Fasting plasma glucose; SBP = Systolic blood pressure; DBP = Diastolic blood pressure; LDL = Low density lipoprotein; HDL = High density lipoprotein; TG = Triglyceride; eGFR = estimated Glomerulus filtration rate; ASCVD = Atherosclerotic cardiovascular disease
