# Supplementary material for: Glycaemic, cardiorenal, and lipid parameters associated with SGLT2 inhibitors use in Indonesian patients with type 2 diabetes: 12-month multicenter real-world study
Source: PLoS One. 2026 Jul 17;21(7):e0353564. doi: 10.1371/journal.pone.0353564 (PMC13378966; doi:10.1371/journal.pone.0353564)
Supplement: S5 Table — (DOCX) [file pone.0353564.s005.docx]

**S5. Full logistic regression output**

| **Outcome** | **Median** | **OR** | **95% CI** | **p-value** | **Covariates adjustment^a^** |
| --- | --- | --- | --- | --- | --- |
| HbA1c | -0.5% | 1.855 | 1.083 – 3.179 | 0.024 | Age, gender, diabetes duration, the quantity of antidiabetic agents, history of coronary artery disease (CAD), history of hypertension, history of diabetic neuropathy, baseline BMI, baseline HbA1c, and baseline GLP-1 receptor agonist therapy. |
| FPG | -12 mg/dL | 2.000 | 1.070 – 3.739 | 0.030 | Age, gender, diabetes duration, the quantity of antidiabetic agents, baseline BMI, and baseline FPG. |
| Body weight | -0.4 kg | 1.756 | 1.191 – 2.589 | 0.004 | Age, gender, history of coronary artery disease (CAD), history of hypertension, and baseline body weight. |
| BMI | -0.15 kg/m^2^ | 1.606 | 1.068 – 2.415 | 0.023 | Age, gender, diabetes duration, and baseline BMI. |
| SBP | -2 mmHg | 1.294 | 0.809 – 2.069 | 0.283 | Age, gender, history of coronary artery disease (CAD), history of stroke, history of diabetic nephropathy, baseline BMI, and baseline SBP. |
| DBP | 0 mmHg | 1.345 | 0.862 – 2.098 | 0.192 | Age, gender, history of coronary artery disease (CAD), baseline BMI, and baseline DBP. |
| LDL-C | -6 mg/dL | 2.070 | 1.255 – 3.414 | 0.004 | Age, gender, diabetes duration, history of dyslipidemia, baseline BMI, and baseline LDL. |
| HDL-C | 1 mg/dL | 1.229 | 0.746 – 2.025 | 0.418 | Age, gender, history of dyslipidemia, baseline BMI, and baseline HDL. |
| Triglycerides | -12 mg/dL | 0.722 | 0.433 – 1.206 | 0.214 | Age, gender, smoking status, diabetes duration, baseline BMI, and baseline triglycerides. |
| Total cholesterol | -7 mg/dL | 1.131 | 0.643 – 1.990 | 0.668 | Age, gender, smoking status, history of coronary artery disease (CAD), history of stroke, baseline BMI, and baseline total cholesterol. |
| eGFR | 0 mL/minute/1,73 m^2^ | 1.803 | 1.048 – 3.100 | 0.033 | Age, gender, the quantity of antidiabetic agents, baseline BMI, and baseline eGFR. |
| ASCVD risk | -0.2% | 1.425 | 0.837 – 2.427 | 0.192 | Age, gender, smoking status, baseline BMI, and baseline ASCVD risk. |

**^a^**Covariates adjustment based on the p-value for each variable (p<0.25).

**^b^**HbA1c = glycated hemoglobin; BMI = Body mass index; FPG = Fasting plasma glucose; SBP = Systolic blood pressure; DBP = Diastolic blood pressure; LDL = Low density lipoprotein; HDL = High density lipoprotein; TG = Triglyceride; eGFR = estimated Glomerulus filtration rate; ASCVD = Atherosclerotic cardiovascular disease.
